# P2RX1 promotes mitochondrial apoptosis via calcium/CaM KII-mediated suppression of PI3K/Akt signaling in Philadelphia chromosome-positive acute lymphoblastic leukemia

**DOI:** 10.3389/fped.2025.1730429

**Published:** 2025-12-12

**Authors:** Xiao bing Li, Qin Ren, Xiang mei Ye, Jing lie Li, Lei guang Feng

**Affiliations:** 1 Department of Laboratory Diagnostics, The First Affiliated Hospital of Harbin Medical University, Harbin, China; 2 Central Laboratory of the First Affiliated Hospital of Harbin Medical University, Heilongjiang Provincial Institute of Hematology & Oncology, Harbin, China

**Keywords:** Ph^+^ ALL, P2RX1, CaMKII, apoptosis, PI3K/Akt

## Abstract

**Introduction:**

Philadelphia chromosome-positive acute lymphoblastic leukemia (Ph^+^ ALL) continues to pose a major clinical challenge. Although tyrosine kinase inhibitor (TKIs)-based regimens form the cornerstone of therapy, treatment outcomes are compromised by difficulties in achieving remission, high relapse rates, and the critical issue of drug resistance. This study aims to elucidate the role of the purinergic receptor P2RX1 in the pathogenesis of and therapeutic response in Ph^+^ ALL.

**Methods:**

By analyzing P2RX1 expression levels in an online patient database and its association with prognosis, we evaluated the potential significance of this receptor in clinical outcomes. A P2RX1 overexpression model was established in the SUP-B15 Ph^+^ ALL cell line, and a series of functional experiments were conducted to assess its impact on tyrosine kinase inhibitor-induced apoptosis and proliferation capacity. To further elucidate its mechanism of action, we monitored changes in intracellular calcium ion concentration, mitochondrial membrane potential, and ATP production levels. Additionally, RT-PCR and Western blot analysis were employed to assess the activation status of the PI3K/Akt signaling pathway, CaMKII, and apoptosis-related proteins BAX, BAD, cytochrome C, and caspases

**Results:**

Analysis of the online patient database revealed that high expression of P2RX1 was significantly associated with poor clinical outcomes. Functional experiments demonstrated that overexpression of P2RX1 in SUP-B15 cells markedly enhanced their sensitivity to apoptosis induced by tyrosine kinase inhibitors; conversely, treatment with the CaMKII inhibitor KN-62 significantly suppressed cell proliferation. Mechanistically, excessive P2RX1 activation disrupts intracellular calcium homeostasis, leading to reduced mitochondrial membrane potential and ATP depletion, thereby activating the intrinsic apoptotic pathway. This process involves inhibition of the PI3K/Akt signaling pathway, hyperactivation of CaMKII, and upregulation of pro-apoptotic proteins such as BAX, Bad, cytochrome C, and cleaved caspase-3 and cleaved caspase-9.

**Conclusion:**

This study reveals the role of P2RX1 as a calcium-regulated tumor suppressor in Ph^+^ ALL, promoting apoptosis by disrupting mitochondrial function and inhibiting the PI3K/Akt survival signaling pathway, thereby providing a novel therapeutic approach to overcome TKI resistance.

## 1 Introduction

The Philadelphia (Ph) chromosome, specifically the *t*(9; 22) (q34; q11) translocation, has been identified as the most prevalent cytogenetic abnormality in patients diagnosed with acute lymphoblastic leukemia (ALL) (1, 2). Approximately 25% of adults diagnosed with Precursor B-cell lymphoblastic leukemia (B-ALL) subsequently develop Ph-positive (Ph^+^) ALL, a condition characterized by the formation of the BCR-ABL1 fusion gene on the Ph chromosome (3). This gene encodes a tyrosine kinase whose dysregulated activity ultimately leads to leukemia (4). Philadelphia chromosome-positive acute lymphoblastic leukemia (Ph^+^ ALL) has long been considered the most poor-prognosis leukemia due to the poor efficacy of conventional multi-agent chemotherapy. BCR-ABL1 protein-specific tyrosine kinase inhibitors (TKIs) have been identified as playing a pivotal role in the treatment of Ph^+^ ALL (5). Regrettably, resistance to tyrosine kinase inhibitors (TKIs) is a common occurrence in Ph^+^ ALL, and the prognosis for these patients remains unfavorable (6, 7). Therefore, it is imperative to identify new therapeutic targets.

The purinergic signaling system, comprising receptors for extracellular nucleotides and nucleosides, has emerged as a key player in cancer pathophysiology (8–10). This system is divided into P1 receptors, which are G-protein coupled receptors for adenosine, and P2 receptors, which are activated by ATP and other nucleotides. The P2 receptor family is further categorized into metabotropic P2Y receptors and ionotropic P2X receptors. Among them, the P2X7 receptor has garnered significant attention in oncology for its role in inflammation and cell death (11–13). Prior research on the P2X receptor in leukemia suggests a multifaceted role, as its overexpression has been positively correlated with non-remission rates and poor prognosis (14). In contrast, as a natural agonist of purinergic receptors, ATP inhibits U-937 cell proliferation in a dose-dependent manner (15). Low micromolar concentrations of ATP trigger differentiation toward a more mature phenotype via P2 purinergic receptors, while higher concentrations are cytotoxic. Other studies have shown that the addition of ATP to the chemotherapeutic agent cytarabine boosts anti-leukemic activity and mitigates matrix-mediated chemotherapy resistance (16). In acute myeloid leukemia, extracellular adenosine and adenosine monophosphate suppress THP-1 cell growth by inducing apoptosis and inhibiting cell cycle arrest, ultimately leading to cell death and modulating SDF-1/CXCR4 axis function (17). Research on its inhibitors reveals that P2X1 antagonists fully suppress AML cell proliferation (18). Similarly, the Jurkat leukemia cell line displays high intracellular ATP concentrations and produces elevated extracellular ATP. Results indicate that purinergic signaling amplified via P2X1 and P2X7 receptors increases baseline cytoplasmic calcium ion (Ca²^+^) levels in Jurkat cells. Inhibition of this basal purinergic signaling mechanism diminishes mitochondrial function, calcium signaling, and cell proliferation. Similar findings were noted in THP-1, U-937, and HL-60 cells. The combination of P2X1 or P2X7 receptor inhibitors with the chemotherapeutic agent 6-mercaptopurine completely halted Jurkat cell proliferation (19). However, the closely related P2RX1 receptor, an ATP-gated calcium channel, has been comparatively overlooked despite its documented expression on normal and malignant B lymphocytes (20, 21). Critically, our preliminary study from Ph^+^ ALL patient cohorts revealed that low expression of P2RX1 is significantly associated with inferior prognosis (22), suggesting that P2RX1 may function as a tumor suppressor in this disease.

Functionally, upon ATP binding, P2RX1 opens to permit a rapid influx of cations, particularly calcium (Ca²^+^), into the cell. This surge in intracellular Ca²^+^ is a potent second messenger. Critically, mitochondria, as central regulators of cellular metabolism and apoptosis, actively uptake Ca²^+^. While moderate Ca²^+^ signaling supports cellular functions, sustained or excessive Ca²^+^ load can trigger the mitochondrial permeability transition pore (MPTP) opening, a point-of-no-return in the intrinsic apoptosis pathway (23–26). MPTP opening leads to the release of pro-apoptotic factors like cytochrome c, initiating a caspase cascade that executes programmed cell death. The calcium/calmodulin-dependent protein kinase II (CaMKII) has been identified as a key mediator translating Ca²^+^ signals into this apoptotic response (27, 28). Therefore, based on our clinical observation of its prognostic value, we hypothesized that P2RX1 activation could exploit this Ca²^+^-mitochondria-apoptosis axis to exert anti-leukemic effects in Ph^+^ ALL.

This study investigated the tumor-suppressive role of P2RX1 in Ph^+^ ALL. We analyzed patient expression data from public databases and overexpressed P2RX1 in SUP-B15 cells to examine its functional impact. Through RNA sequencing assays combined with functional assays assessing proliferation, apoptosis, mitochondrial function, and metabolism, we identified key downstream molecular pathways influenced by P2RX1, revealing its potential as a therapeutic target in Ph^+^ ALL.

## 2 Results

### 2.1 P2RX1 plays a vital role in ALL

**Figure 1:**
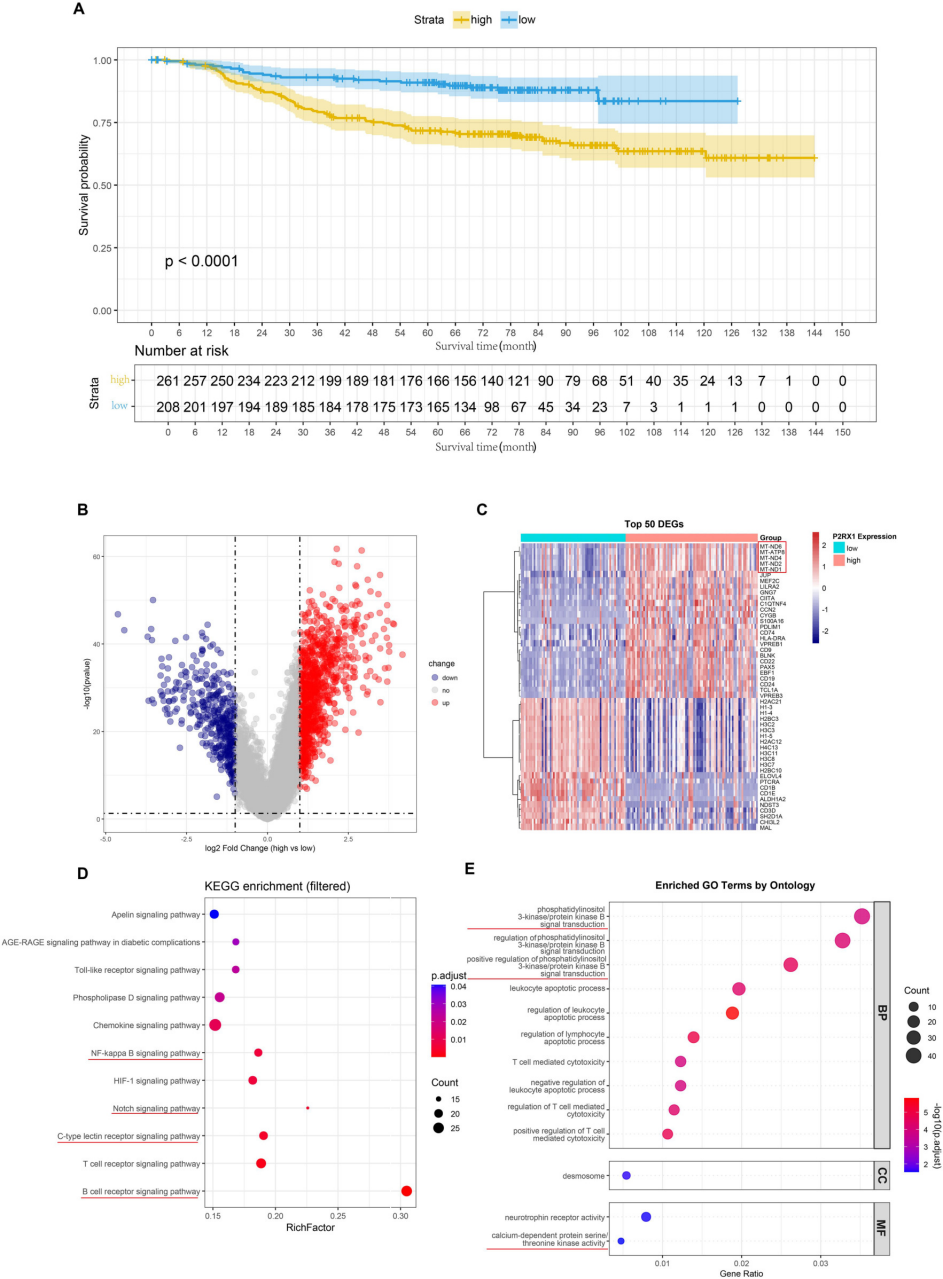
Overall survival curve **(A)**, volcano plot of differentially expressed genes between groups **(B)**, visualization of top 50 differentially expressed genes **(C)**, KEGG enrichment analysis of differentially expressed genes filtered by specific terms **(D)**, GO enrichment analysis of differentially expressed genes filtered by specific terms **(E)**. Source: KEGG (https://www.kegg.jp/, accessed July 2025), © Kanehisa laboratories (38).

A total of 532 patient clinical follow-up records and sequencing data were obtained from TARGET-ALL-P2, including 469 distinct patients diagnosed with acute lymphocytic leukemia (ALL) or precursor B-cell lymphoblastic leukemia (B-ALL). The subsequent analysis involved a comparison of overall survival (OS) between the two groups (Figure 1A). A substantial disparity in survival duration was evident between the groups (*p* < 0.0001), with the P2RX1 high-expression group demonstrating a reduced survival probability compared to the low-expression group. The differential expression of genes (DEGs) between these groups was subsequently analyzed and visualized. As illustrated in Figure 1B, the volcano plot of DEGs reveals that blue corresponds to downregulated genes, red indicates upregulated genes, and gray designates genes with non-significant changes. As illustrated in Figure 1C, the top 50 most significantly altered genes are displayed, with all genes circled in red indicating mitochondrial-related genes. Subsequently, KEGG and GO enrichment analyses were performed on all differential genes (Figures 1D,E). Following the filtration of the results, the most salient signaling pathway identified was the B-cell receptor signal pathway, with its primary downstream pathway being the PI3K/Akt signaling pathway. GO enrichment analysis results indicate that biological processes such as calcium ion metabolism and positive regulation of 3-phosphokinase are enriched.

### 2.2 P2RX1 overexpression and RNA expression profile sequencing

**Figure 2:**
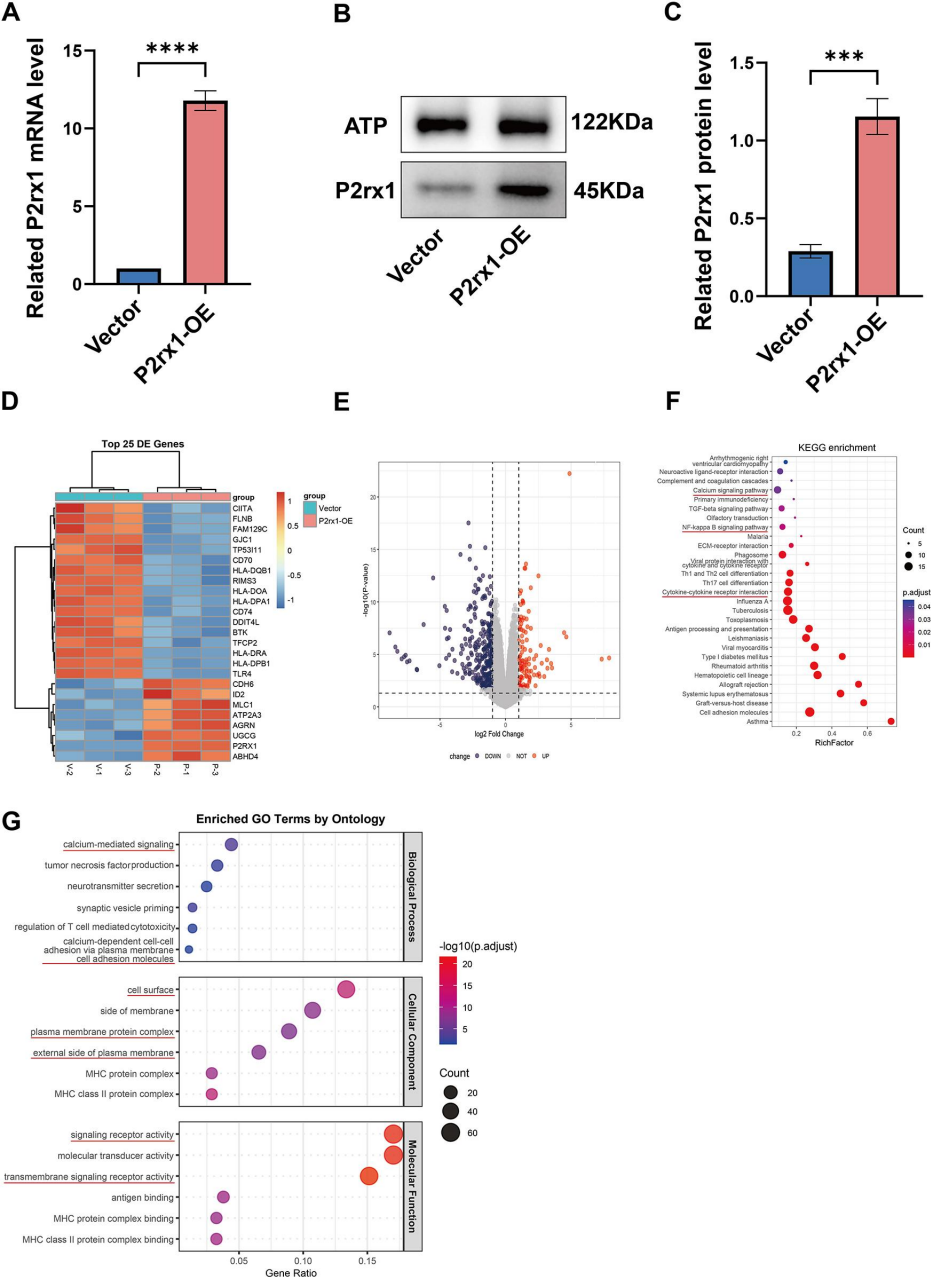
Real-time quantitative fluorescent PCR analysis of P2RX1 gene mRNA expression between two groups **(A)**, analysis of P2RX1 protein expression changes using the sodium-potassium ATP pump on the membrane as an internal reference **(B)**, and statistical analysis **(C)**. Sequencing data underwent differential gene analysis between groups **(D)** and visualization **(E)**, followed by KEGG enrichment analysis of differential genes **(F)**. GO enrichment analysis **(G)**. Experiments were independently replicated three times. Data are presented as mean ± standard deviation; *P < 0.05; **P < 0.01; ***P < 0.001; ****P < 0.0001; ns indicates no significant difference. Source: KEGG (https://www.kegg.jp/, accessed July 2025), © Kanehisa Laboratories (38).

Following lentiviral transduction of SUP-B15 cells, successful transduction was confirmed by RT-PCR. Analysis revealed a significant difference in mRNA expression levels between the empty vector group and the target gene construct group (*p* < 0.0001) (Figure 2A). This finding was further validated at the protein level by western blot analysis (Figure 2B). Using sodium-potassium ATPase (Na^+^/K^+^-ATPase) as a loading control, quantitative analysis indicated that P2RX1 protein1 protein expression was approximately threefold higher in the P2RX1-overexpressing (P2RX1-OE) group compared to the vector control group; this difference was statistically significant (*p* < 0.001) (Figure 2C). Subsequently, RNA sequencing (RNA-seq) was performed on both groups to identify differentially expressed genes (DEGs). A heatmap visualizing the top 25 most significantly altered genes (Figure 2D) and a volcano plot displaying all DEGs (Figure 2E) were generated. Pathway enrichment analyses using the KEGG and GO databases revealed several significantly enriched terms (Figures 2F,G). These included pathways related to calcium ion signaling, plasma membrane receptor complexes, and NF-*κ*B signaling, which were consistent with findings from established online repositories.

### 2.3 Effects of P2RX1 gene overexpression on proliferation

The CCK-8 assay was employed to determine appropriate drug concentrations, as shown in Supplementary Figures. Supplementary Figure S1 shows the concentration screening process for KN-62, based on which 10 μM was selected as the effective inhibitory concentration. Similarly, Supplementary Figure S2 presents the screening process for imatinib, leading to the selection of 20 μM as the working concentration.

**Figure 3:**
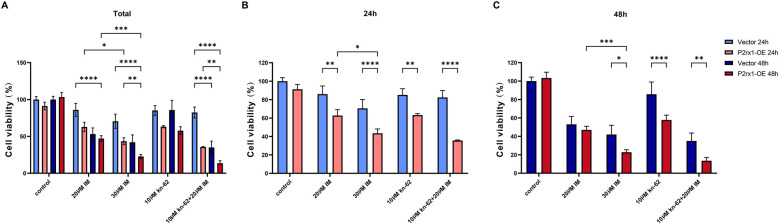
Relative cell viability plots. Total plots at 24 and 48 hours **(A)**, 24 hour plot **(B)**, and 48 hour plot **(C)** Experiments were independently repeated three times. Data are expressed as mean ± standard deviation; *P < 0.05; **P < 0.01; ***P < 0.001; ****P < 0.0001; ns indicates no significant difference.

Treatment with either 20 μM or 30 μM imatinib in different cell groups, or with 10 μM KN-62 for 24 and 48 h (Figure 3), effectively suppressed cell proliferation in a time- and dose-dependent manner. Under identical drug concentrations and treatment durations, the P2RX1-OE group exhibited a significantly higher inhibition rate of cell proliferation than the Vector group (*P* < 0.01), indicating that P2RX1-overexpressing cells were more susceptible to proliferation inhibition. Furthermore, the strongest suppression of cell viability was observed in the combination treatment group receiving 20 μM imatinib along with 10 μM KN-62.

### 2.4 Effects of P2RX1 gene overexpression on apoptosis

**Figure 4:**
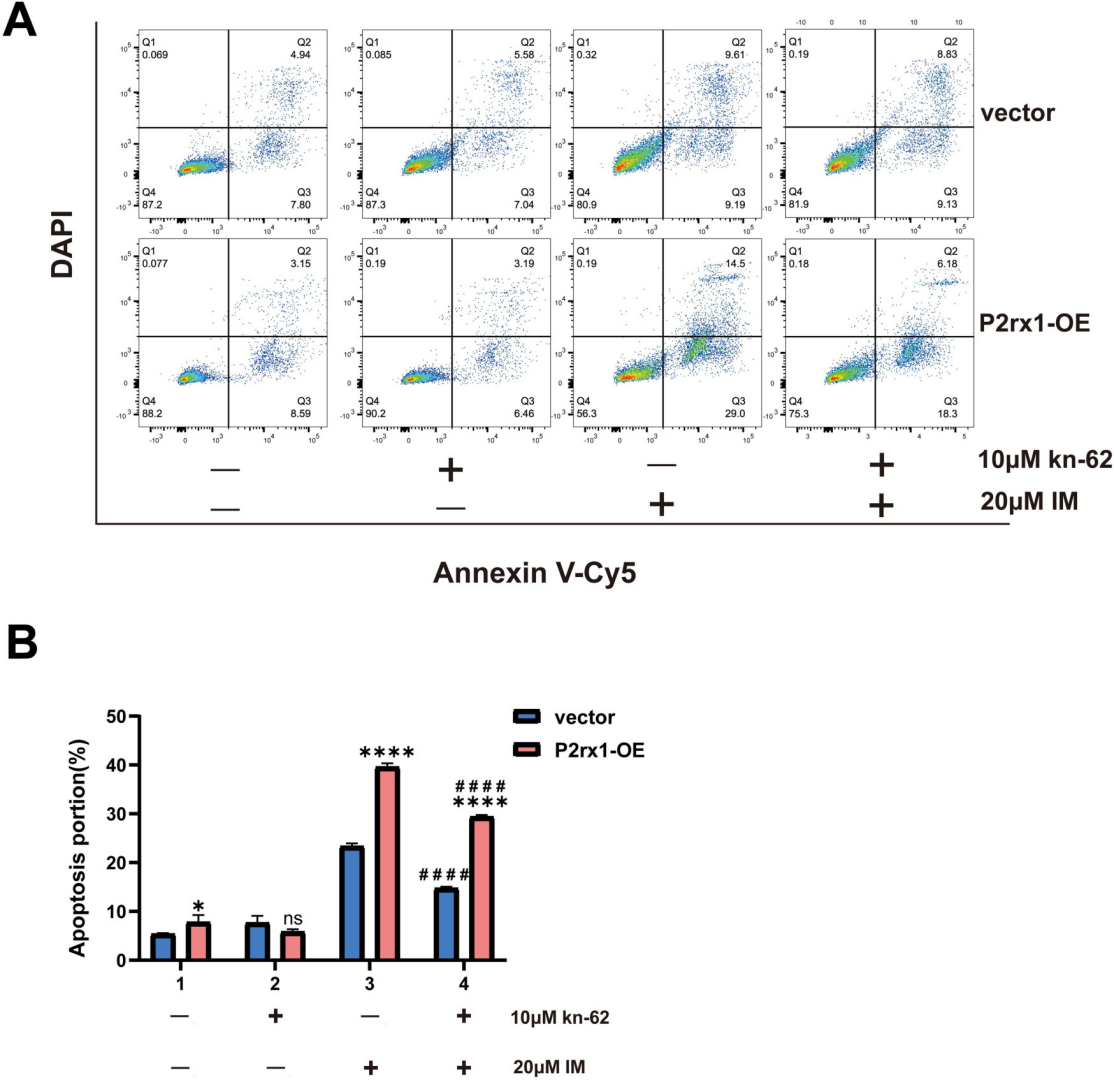
Representative flow cytometry dot plot **(A)** and statistical analysis bar chart **(B)**. Experiments were independently replicated three times. Data are expressed as mean ± standard deviation. *indicates comparison between the P2RX1-oe group and the corresponding vector control group; # indicates comparison between the dual-drug group and the corresponding single-drug 20 μM imatinib group. *P < 0.05; **P < 0.01; ***P < 0.001; ****P < 0.0001; ns indicates no significant difference.

Using membrane protein V, Cy5/DAPI dual staining, the P2RX1-OE group demonstrated significantly increased proportions of both early and late apoptotic cells compared to the vector control group following treatment with 20 μM imatinib (*P* < 0.0001; Figures 4A,B). In contrast, no statistically significant pro-apoptotic effect was observed in the 10 μM KN-62 monotherapy group (ns.). When treated with the combination of 20 μM imatinib and 10 μM KN-62, a marked reduction in apoptotic cells occurred relative to 20 μM imatinib alone, indicating that inhibition of CaMKII activity during apoptosis induction can partially rescue cell death. A statistically significant difference was found both between the two monotherapy groups (*P* < 0.001) and between the two combination therapy groups (*P* < 0.001). These results suggest that differential expression levels of the P2RX1 gene influence cellular susceptibility to apoptosis induction, with the P2RX1-OE group being more readily triggered to undergo apoptosis.

### 2.5 Overexpression of the P2RX1 gene alters intracellular calcium ion levels and energy metabolism

**Figure 5:**
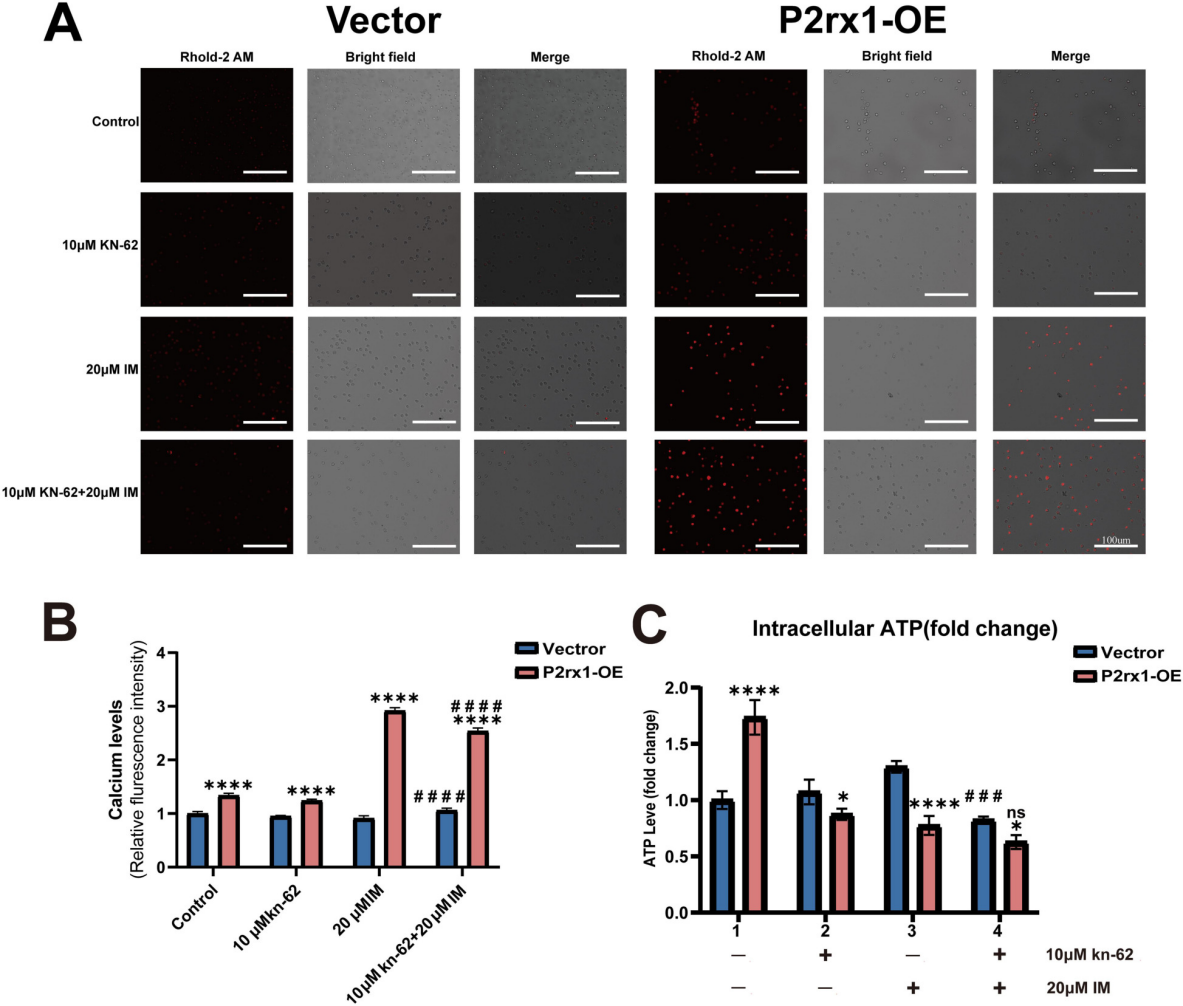
Photograph after Rhod-2 AM staining, scale bar 100 μm **(A)**; quantitative analysis of calcium ion level changes **(B)**; quantitative analysis of intracellular ATP changes **(C)**. Experiments were independently repeated three times. Data are expressed as mean ± standard deviation. *indicates comparison between the P2RX1-oe group and the corresponding vector control group; # indicates comparison between the dual-drug group and the corresponding single-drug 20 μM imatinib group; *P < 0.05; **P < 0.01; ***P < 0.001; ****P < 0.0001; ns indicates no significant difference.

Both groups were subjected to intracellular calcium staining using Rhod-2 AM, which revealed significantly higher calcium levels in the P2RX1-OE group relative to the vector control group. Treatment with 20 μM imatinib markedly increased intracellular calcium in the P2RX1-OE group (*P* < 0.0001; Figures 5A,B), while 10 μM KN-62 alone did not induce noticeable changes. Co-administration of both drugs significantly attenuated intracellular calcium levels compared with 20 μM imatinib alone (*P* < 0.0001); however, these levels remained significantly elevated compared with the control group. These findings indicate that Ca²^+^ overload plays a critical role in the apoptosis induction process.

Measurement of intracellular ATP levels showed that (Figure 5C), under baseline conditions, the P2RX1-OE group exhibited significantly higher ATP levels than the vector group (*P* < 0.001). Upon monotherapy with either agent, ATP levels in the P2RX1-OE group decreased significantly compared with the corresponding vector-treated group (*P* < 0.05). Following combination therapy, the reduction in ATP levels was more pronounced in the P2RX1-OE group than in the vector group (*P* < 0.05), though this decrease was not statistically different from that observed with 20 μM imatinib alone. Together, these data suggest that mitochondrial function, mitochondrial function, and metabolic capacity are compromised during the process.

### 2.6 Overexpression of the P2RX1 gene could induce alterations in mitochondrial function

**Figure 6:**
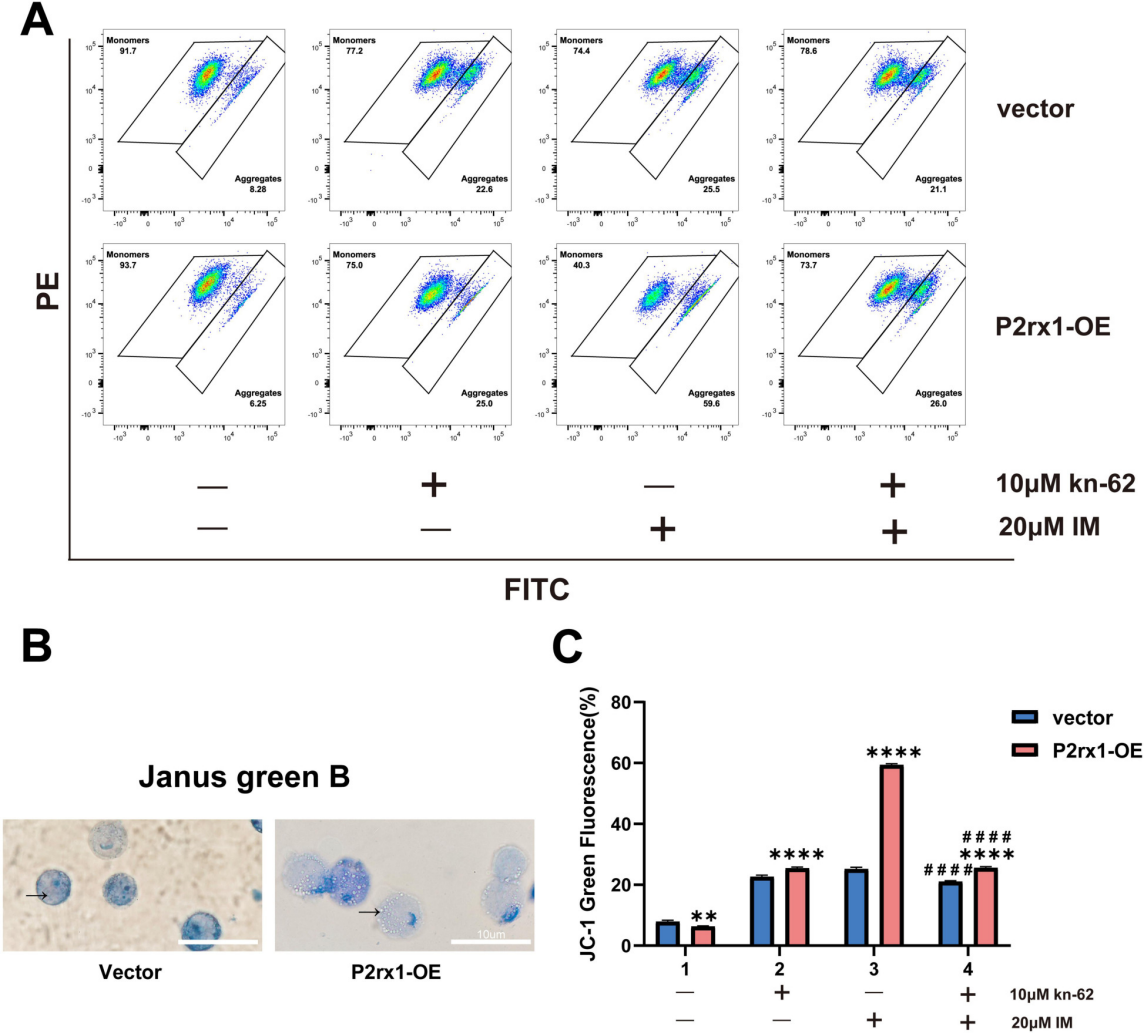
Overexpression of P2RX1 reduced mitochondrial membrane potential (MMP) in SUP B15 cells. MMP was measured by flow cytometry using JC-1 staining. Red fluorescence (PE channel) represents polymerized forms, while green fluorescence (FITC channel) represents monomeric forms **(A,C)**. Mitochondrial changes following Janus Green B staining are indicated by arrows **(B)**. Experiments were independently repeated three times. Data are expressed as mean ± standard deviation. *indicates comparison between the P2RX1-oe group and the corresponding vector control group; # indicates comparison between the dual-drug group and the corresponding single-drug 20 μM imatinib group; *P < 0.05; **P < 0.01; ***P < 0.001; ****P < 0.0001; ns indicates no significant difference.

JC-1 staining indicated that both 10 μM KN-62 and 20 μM imatinib significantly reduced the mitochondrial membrane potential (ΔΨm) compared with the control group (*P* < 0.05). The most substantial decrease in ΔΨm was observed in the 20 μM imatinib monotherapy group, which differed significantly from the vector control group (*P* < 0.001). Notably, the combination of imatinib and KN-62 led to a less pronounced reduction in ΔΨm than imatinib alone. Moreover, under combination treatment, the loss of ΔΨm remained more prominent in the P2RX1-overexpressing group than in the vector group (*P* < 0.001; Figures 6A,C). Janus Green B staining revealed considerable mitochondrial swelling mitochondrial swelling in the overexpression groups (Figure 6B, arrows), further supporting the notion that mitochondrial structural integrity is compromised. Taken together with earlier data on intracellular Ca²^+^ dys^+^ dysregulation, these results strongly suggest that mitochondrial dysfunction plays a central role in the apoptotic process.

### 2.7 Overexpression of the P2RX1 gene may increase endogenous apoptosis by inhibiting the PI3K/Akt pathway

**Figure 7:**
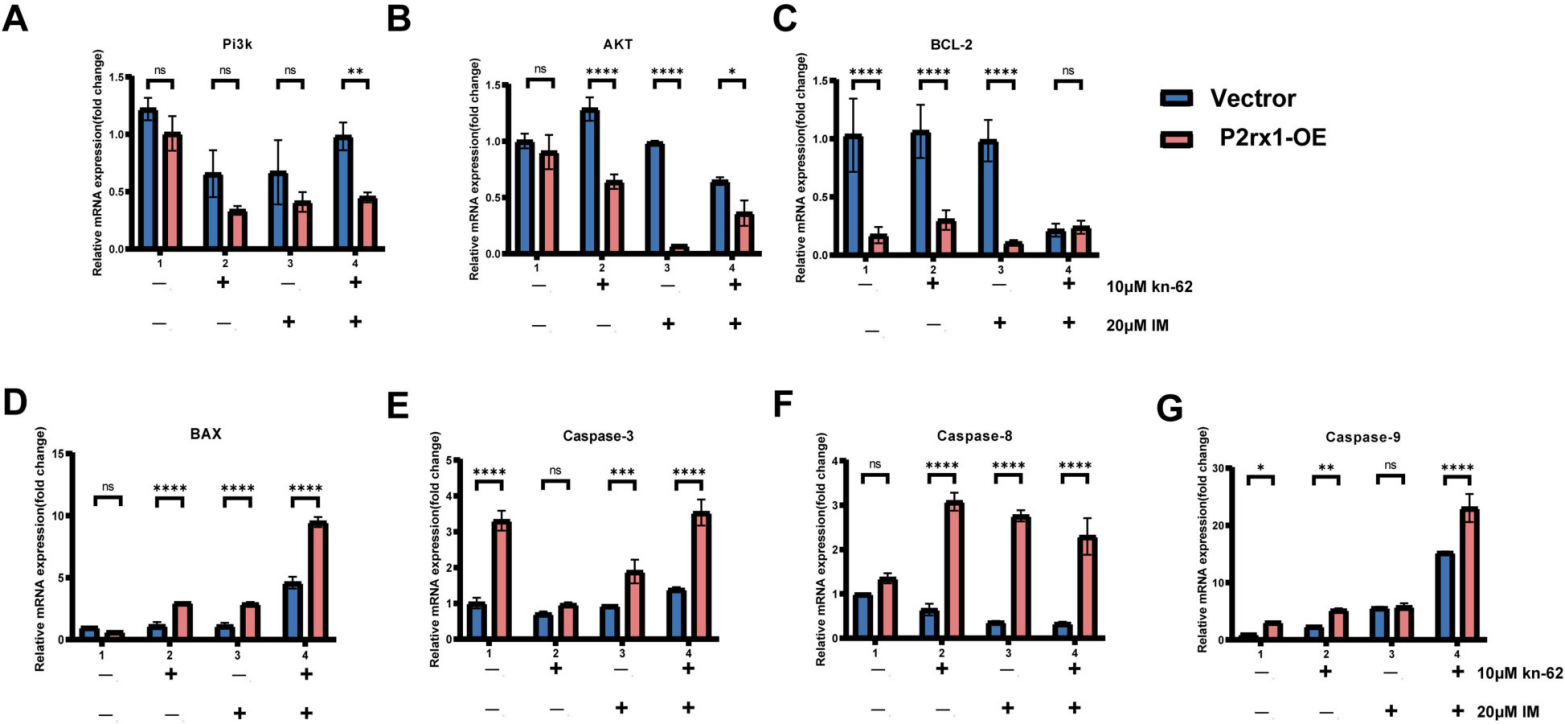
Analysis of real-time quantitative PCR results for fluorescence detection: Pi3k **(A)**, AKT **(B)**, BCL-2 **(C)**, BAX **(D)**, caspase-3 **(E)**, caspase-8 **(F)**, caspase-9 **(G)**. **(E)**, caspase-8 **(F)**, and caspase-9 **(G)**. Experiments were independently replicated three times. Data are presented as mean ± standard deviation. Symbols indicate statistical significance: *P < 0.05; **P < 0.01; ***P < 0.001; ****P < 0.0001; ns indicates no significant difference.

Based on our previous findings, we next sought to explore key regulatory molecules within the intrinsic apoptosis pathway. Real-time quantitative PCR analysis revealed that, under matched drug treatments, the mRNA expression levels of PI3K and Akt were significantly downregulated in the combination therapy group compared to the control group. This suppression was particularly pronounced when compared to the identically treated empty vector group (*P* < 0.01; Figure 7). Concurrently, transcript levels of core pro-apoptotic factors were markedly elevated (*P* < 0.001) relative to both the untreated control and the dose-matched vector control group.

**Figure 8:**
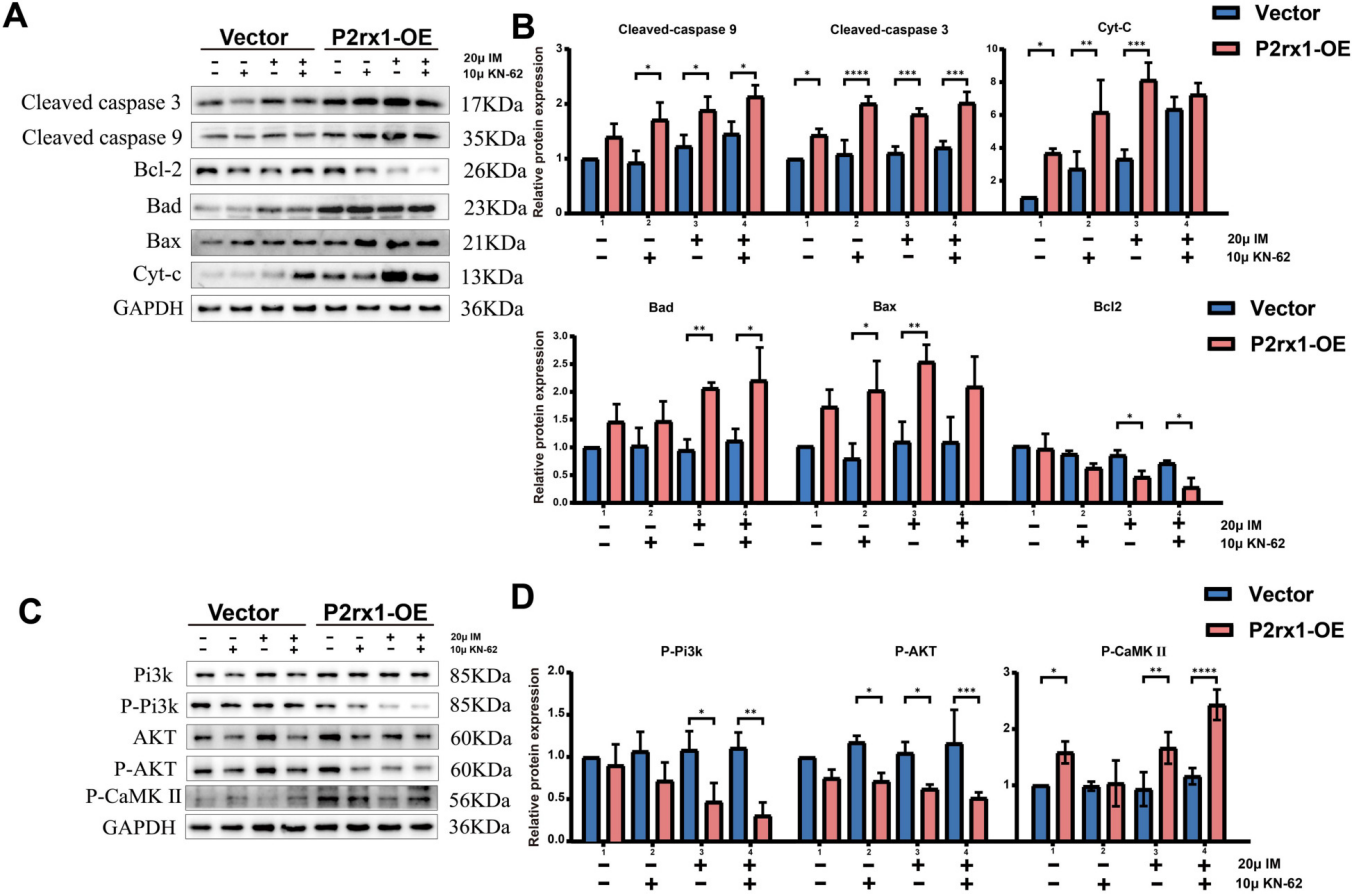
WB experiments validate endogenous apoptosis-related molecular expression **(A)** and statistical analysis **(B)**, Pi3k/AKT signaling pathway and P-CaM KIImolecular expression changes **(C)**, and statistical analysis **(D)**. Experiments were independently repeated three times. Data are presented as mean ± standard deviation. *P < 0.05; **P < 0.01; ***P < 0.001; ****P < 0.0001; ns indicates no significant difference.

This result was validated by Western blot (WB) analysis. Under identical treatment conditions, P2RX1-overexpressing cells showed significantly diminished phosphorylation of PI3K and AKT (*P* < 0.05; Figure 8) but a notable increase in P-CaMKII levels (*P* < 0.01) relative to the vector control group. Additionally, the P2RX1-OE group exhibited substantially upregulated expression of several pro-apoptotic proteins—BAX, Bad, cytochrome c (Cyt-C), cleaved caspase-9, and cleaved caspase-3—alongside a pronounced downregulation of the anti-apoptotic protein BCL-2. Remarkably, upon treatment with 20 µM imatinib, Cyt-C expression in P2RX1-OE cells was approximately eightfold higher than in vector controls (*p* < 0.001), highlighting robust engagement of the mitochondria-dependent apoptotic pathway.

## 3 Discussion

Previous studies have demonstrated that P2X receptors (P2Xs) function as cation-selective channels, permitting the influx of sodium ions (Na^+^), potassium ions (K^+^), or calcium ions (Ca²^+^). Furthermore, P2Xs are widely expressed in solid tumors and hematologic malignancies (18). The P2X family comprises seven members (P2RX1-7), each exhibiting distinct ion channel properties and functions in response to ATP stimulation. For instance, P2RX1-4 are sensitive to extracellular ATP at nanomolar or micromolar concentrations, whereas P2X7 responds only to millimolar ATP levels (29). P2X receptors perform multiple functions in both normal hematopoiesis and hematologic disorders; for example, P2RX1 and P2X4 may regulate the multipotent fate of hematopoietic stem cells (HSCs) (30). In the lymphoblastic leukemia cell line Jurkat, ATP-induced tension generation and autocrine stimulation of P2RX1 and P2X7 have been observed to increase calcium influx and mitochondrial metabolism, thereby promoting cell proliferation (19). Conversely, some studies have demonstrated that extracellular ATP can inhibit cancer cell proliferation (31). This contradiction may stem from differences in the expression levels of P2 receptor subtypes across various anatomical tissues (9). This study's findings, based on an analysis of Target-ALL-P2 data, indicate that high P2RX1 gene expression is associated with reduced overall survival rates in patients with ALL. This suggests that P2RX1 may serve as a prognostic biomarker for overall survival in this patient population. However, the influence of P2RX1 on apoptosis in ALL cells, as well as the underlying mechanisms involved, remains to be elucidated. Consequently, an *in vitro* model of SUP B15 cells overexpressing P2RX1 was established, and conducted RNA sequencing. Enrichment analysis using online databases (KEGG and GO) revealed significant enrichment in calcium ion regulation and protein phosphorylation pathways.

The regulation of intracellular calcium metabolism is a complex process that involves multiple organelles and structures within the cell. Mitochondria have been shown to regulate calcium release directly (25). Additionally, they have been observed to regulate calcium metabolism indirectly through the modulation of intracellular ATP levels (32). CaM KII activity, on the other hand, is found to be directly regulated by intracellular calcium levels (27). In this study, under identical drug exposure, the P2RX1-OE group exhibited significantly higher calcium levels than the vector group, along with markedly elevated phosphorylated CaM KII levels. Concurrently, the P2RX1-OE group demonstrated a greater reduction in mitochondrial membrane potential and a corresponding decrease in intracellular ATP levels. Furthermore, the previous studies have shown that KN-62 (a CaMKII inhibitor) can inhibit cell proliferation (28). However, compared to imatinib monotherapy, the combination of these two drugs reduced apoptosis levels—a phenomenon potentially attributable to KN-62's inhibition of hyperactivated CaM-KII. This finding indicates that regulation of CaM KII activity plays a critical role in the apoptotic process observed in the P2RX1-OE group.

The PI3K/AKT signaling pathway is a principal mechanism that stimulates cell proliferation and inhibits cell death (33). A wealth of research has revealed this pathway's pivotal role in the onset and progression of various cancers, such as breast, lung, and colorectal cancers (34, 35). Thus, the inhibition of the PI3K/AKT pathway is a primary therapeutic target in cancer treatment (36). Under consistent treatment conditions, our study found notably reduced levels of PI3K and AKT protein phosphorylation in the P2RX1-OE group compared to the Vector group. Simultaneously, there was a significant upregulation of mitochondrial-induced endogenous pro-apoptotic proteins like cytochrome c, cleaved caspase-3, cleaved caspase-9, Bax, and Bad. Remarkably, Cyt-c expression saw an approximate eightfold increase in the 20 μM imatinib group compared to the untreated control group. Conversely, the anti-apoptotic protein Bcl-2 expression was substantially downregulated. This suggests that overexpression of P2RX1 may alter mitochondrial metabolism, leading to calcium instability, excessive CaM KII activation, and inhibition of the PI3K/AKT pathway. The ultimate consequence of these alterations is an increased susceptibility of SUP B15 cells to apoptosis. However, further investigation is required to understand the specific molecular mechanisms involved.

## 4 Materials and methods

### 4.1 Online database data analysis

Clinical follow-up information and sequencing data for patients in the TARGET-ALL-P2 dataset were retrieved using the TCGAbiolinks R package “GDCquery” (version 2.36.0) from the GDC website (https://gdc.cancer.gov). Genomic Data Commons phs000218 (2018). https://portal.gdc.cancer.gov/projects/TARGET-ALL-P2. Patients were grouped based on P2RX1 gene expression TPM values, which were calculated using the optimal intercept method. The cutoff point for P2RX1 gene expression TPM was set at 41.4635, thereby dividing patients into low-expression and high-expression groups. The generation of overall survival curves was accomplished by employing the ggsurvplot function within the survminer R package. P-values were calculated using the log-rank test. The limma package (version 3.58.1) was utilized to evaluate the disparities in gene expression between the two cohorts. The selection of differentially expressed genes (DEGs) was based on the criteria of |logFC| > 1 and an adjusted *p*-value < 0.05. The Benjamini-Hochberg (BH) method was employed for *p*-value correction. Volcano plots for differential analysis were generated using the ggplot2 package (version 3.4.4). A heatmap of the top 50 differentially expressed genes (DEGs) was generated using the R package “heatmap” (version 1.0.12). GO and KEGG enrichment analyses were performed on all DEGs using the clusterProfiler package (version) in R, with *p*-values calculated based on the hypergeometric distribution.

### 4.2 Cell culture and transduction

SUP-B15 cells were obtained from Zhejiang Baidi Biotechnology Co., Ltd. The cells were cultured in Iscove's Modified Dulbecco's Medium (IMDM; Procell, China) supplemented with 20% fetal bovine serum (FBS; CellBox CellBox, USA), 100 U/mL penicillin, and 100 μg/mL streptomycin. Cultures were maintained at 37 °C in a humidified atmosphere containing 5% CO₂ (Thermo Fisher Scientific, USA).Log-phase SUP-B15 cells were seeded into 96-well plates at a density of 1 × 10⁴ cells per well. Cells were then transduced with lentiviral particles (Shanghai GK Gene) carrying either P2RX1 overexpression construct or a nonspecific control (NC) sequence at a multiplicity of infection (MOI) of 60. A transduction enhancer was added at a concentration of 4 µL/mL to improve infection efficiency. After successful transduction, stable cell lines were selected using 5 µg/mL puromycin. All experimental procedures were performed in compliance with relevant guidelines and regulations and were approved by the Institutional Review Board of the Heillongjiang Provincial Blood Cancer Laboratory.

### 4.3 RNA sequencing (RNA-Seq) assay

SUP-B15 cells stably overexpressing P2RX1 (P2RX1-OE) and empty vector controls (EV) were used for RNA sequencing. Total RNA was extracted using Total RNA Extraction Reagent (Vazyme, #R711-01), and its purity and concentration were determined with a NanoDrop2000 spectrophotometer (Thermo Fisher, USA). Qualified RNA samples were subsequently subjected to sequencing on an Illumina NovaSeq6000 platform (Illumina, USA) by Berry Genomics (China). The selection of differentially expressed genes (DEGs) was based on the criteria of |logFC| > 1 and an adjusted *p*-value < 0.05. The Benjamini-Hochberg (BH) method was employed for *p*-value correction. Volcano plots for differential analysis were generated using the ggplot2 package (version 3.4.4). A heatmap of the top 50 differentially expressed genes (DEGs) was generated using the R package “heatmap” (version 1.0.12). GO and KEGG enrichment analyses were performed on all DEGs using the clusterProfiler package (version 4.0.5) in R, with p-values calculated based on the hypergeometric distribution.

### 4.4 Cell counting kit-8 (CCK-8) assay

Cell viability was determined using the CCK-8 assay kit (C0005, TargetMol, Shanghai, China). Cells in the logarithmic growth phase were seeded into 96-well plates at a density of 5 × 10⁴ cells per well in 100 µL of medium. At 24, 48, and 72 h after seeding, 10 µL of CCK-8 reagent was added to each well and incubated at 37 °C for 2 h. The absorbance at 450 nm was measured using a SpectraMax M5 Multifunction Microplate Reader (Molecular Devices, USA), with the absorbance at 650 nm recorded simultaneously for background correction. Cell viability was calculated according to the following formula: Cell Viability (%) = [(A treatment—A blank)/(A control—A blank)] × 100, where A represents the corrected absorbance (OD 450 nm–OD 650 nm). Here, A blank refers to wells containing only culture medium and CCK-8 reagent, and A control represents the untreated control cells.

### 4.5 Quantitative real-time PCR (RT-qPCR)

Total RNA was isolated from cells using Trizol reagent (B610409, Sangon Biotech, Shanghai, China) according to the manufacturer's protocol. We assessed RNA concentration and purity using a NanoDrop 2000 spectrophotometer (Thermo Fisher Scientific, USA). Qualified RNA samples were reverse-transcribed into complementary DNA (cDNA) with a commercial reverse transcription kit (RK20429, ABclonal, Wuhan, China). Quantitative real-time PCR (qPCR) was subsequently performed using synthesized cDNA templates and gene-specific primers. The PCR reaction conditions were as follows: 95 °C for 3 min, followed by 40 cycles, each cycle consisting of 95 °C for 15 s and 56 °C for 1 min. Product specificity was subsequently verified through melt curve analysis using the instrument's default dissociation protocol. The relative expression levels of the target genes were calculated using the 2^−ΔΔCt^ methodCt method, with GAPDH mRNA serving as the internal reference for normalization.

The oligonucleotide primers used in this study were obtained from two sources. Primers for Bcl-2, Bax, Caspase-3, Caspase-8, and Caspase-9 were sourced from previously published literature (37), while those targeting p2rx1 (NM_002558.4), PIK3R1 (NM_181523.3), and AKT1 (NM_001014431.2) were newly designed using Primer Premier 5.0 software. All oligonucleotides were custom-synthesized by ApexBio Technology (Shanghai, China). The sequences of all primers used are provided in Supplementary Table S1.

### 4.6 Apoptosis assay

The apoptotic rate of cells was detected using an Annexin V-Cy5/DAPI apoptosis assay kit (APExBIO, No. K2255). Cells were collected by centrifugation at 300 × g for 5 min at 4 °C. After being washed twice with pre-cooled PBS, the cells were resuspended in 100 μL of 1× Binding Buffer. Subsequently, 2.5 μL of DAPI staining solution and 2.5 μL of Annexin V-Cy5 staining solution were added, and the mixture was gently mixed. The reaction was carried out at room temperature in the dark for 20 min. Then, 400 μL of 1× binding buffer was added to each tube, and the mixture was gently mixed prior to flow cytometry analysis. Samples were analyzed using a BD FACSLyric™ flow cytometer (USA), and data were processed with FlowJo software.

### 4.7 Measurement of mitochondrial membrane potential (MMP)

Mitochondrial membrane potential was assessed using the Enhanced Mitochondrial Membrane Potential Assay Kit (C2003S, Beyotime Biotechnology, Shanghai, China). Treated cells were resuspended in 4 μM JC-1 staining working solution and incubated at 37 °C for 30 min. Following two washes with JC-1 staining buffer, the cells were resuspended in the same buffer and analyzed on a BD FACSLyric™ flow cytometer (USA). Data were processed using FlowJo software.

### 4.8 Intracellular calcium Ion (Ca²^+^) assay

Intracellular calcium levels were measured using Rhod-2, AM probe (MX4507-250UG, Maokangbio, Shanghai, China). Cells were loaded with 5 μM Rhod-2, AM working solution and incubated at 37 °C for 30 min. Fluorescence imaging was performed using an EVOS FL cell imaging system (Thermo Fisher, USA). Parallel fluorescence intensity measurements were conducted with a SpectraMax M5 multifunction microplate reader (Molecular Devices, USA) set at excitation/emission wavelengths of 549/578 nm. Images were analyzed using ImageJ software.

### 4.9 Janus green B staining assay

Mitochondria were stained with Janus Green B (HY-D1122, MCE, USA). USA). A staining working solution was prepared by dissolving 5 μg of Janus Green B in 1.96 mL of mL of PBS to yield a final concentration of 5 μM. Cells were incubated with the working solution for 5 min. Mitochondrial morphology was observed and mitochondria were counted within cells using a standard light microscope.

### 4.10 Determination of ATP levels

Intracellular ATP levels were quantified using an ATP Assay Kit (S0026, Beyotime Biotechnology, Shanghai, China). Cells were collected by centrifugation at 1,500 rpm for 5 min, followed by the addition of 100 μL ATP lysis buffer. The lysates were centrifuged at 12,000 rpm and 4 °C for 5 min, and the resulting supernatant was collected for ATP determination. Both the supernatants and ATP standard solutions were diluted to appropriate concentrations with ATP dilution buffer. Subsequently, 20 μL of each diluted sample or standard was mixed with 100 μL of reaction working solution, and the mixtures were transferred to a black 96-well plate. Luminescence intensity was measured using a SpectraMax M5 Multifunction Microplate Reader (Molecular Devices, USA).

### 4.11 Western blotting analysis

Total protein was extracted from cells using RIPA lysis buffer (P0013B, BiYunTian, Shanghai, China) supplemented with protease and phosphatase inhibitors (K1007, K1015, APExBIO, Houston, USA). Protein concentration was determined with a BCA assay kit (ZJ102, YaEnzyme, Shanghai, China). Then, 20 μg of protein per sample was separated by SDS-PAGE and transferred onto PVDF membranes (IPVH00010, Millipore, Germany). The membranes were incubated with primary antibodies overnight at 4 °C, followed by incubation with corresponding secondary antibodies for 1 h at room temperature. Protein bands were detected using the Omni-ECL™ Ultra-Sensitive Chemiluminescent Detection Kit (SQ201, Yasei, Shanghai, China) and visualized with a Tanon-5200 gel imaging system (Shanghai, China). GAPDH protein was utilized as the internal reference protein. The list of antibodies used can be found in the Supplementary Material (Supplementary Table S2). The grayscale analysis was performed using the ImageJ software.

### 4.12 Statistical analysis

The results were analyzed using GraphPad Prism 9.5.0 and R 4.5.0. Both datasets exhibited normal distribution. When variances were equal, two-tailed paired or unpaired Student's *t*-tests were used to calculate *p*-values between groups. Non-normally distributed data underwent nonparametric analysis. Differences in intergroup variance were analyzed using Welch's correction. Multi-group comparisons were performed using a two-way ANOVA. Survival analysis *p*-values were calculated using the log-rank test. *P*-values were corrected using the Benjamini-Hochberg method, and no data were excluded during the analysis.

## 5 Conclusions

In summary, our findings confirm that P2RX1 inhibits proliferation and promotes apoptosis in SUP B15 cells, potentially expanding the research scope and clinical application prospects of P2RX1. Our mechanistic studies indicate that P2RX1 regulates apoptosis not only through the classical mitochondrial pathway but also via excessive activation of the CaM KIImolecule, particularly by inhibiting the PI3K/AKT signaling pathway. P2RX1 modulates apoptosis through these pathways to maintain intracellular homeostasis, suggesting its potential as a novel target for regulating the progression of Ph^+^ ALL.

## Data Availability

The datasets presented in this study can be found in online repositories. The names of the repository/repositories and accession number(s) can be found below: Mendeley Data, V1, doi: 10.17632/66kbk28dzh.1, li, xiao bing (2025). Accession: Ph+ALL and P2RX1.
