# Supplementary material for: P2RX1 promotes mitochondrial apoptosis via calcium/CaM KII-mediated suppression of PI3K/Akt signaling in Philadelphia chromosome-positive acute lymphoblastic leukemia
Source: Front Pediatr. 2025 Dec 12;13:1730429. doi: 10.3389/fped.2025.1730429 (PMC12741055; doi:10.3389/fped.2025.1730429)
Supplement: Supplementary file 1 [file Supplementaryfile1.docx]

**Supplementary Material**

**Supplementary Table1:**

**Primer sequences for quantitative real-time PCR**

| Gene | Forward | Reverse |
| --- | --- | --- |
| Bcl-2 | 5'-GGCTGGGATGCCTTTGTG-3' | 5'-CAGCCAGGAGAAATCAAACAGA-3' |
| Bax | 5'-TGCTTCAGGGTTTCATCCAG-3' | 5'-GGCGGCAATCATCCTCTG-3' |
| Caspase-3 | 5'-CAGAACTGGACTGTGGCATTG-3' | 5'-GCTTGTCGGCATACTGTTTCA-3' |
| Caspase-8 | 5'-CATCCAGTCACTTTGCCAGA-3' | 5'-GCATCTGTTTCCCCATGTTT-3' |
| Caspase-9 | 5'-CCAGAGATTGCGAAACCAGAGG-3' | 5'-GAGCACCGACATCACCAAATTC-3' |
| PI3K | 5'-CGTAGTCGTGATCGGCGATGCA-3' | 5'-CGTAGCTGGATGCTGATGCGAC-3' |
| AKT | 5'-ACCTGATGCTAGTGCCTGATC-3' | 5'-CTAGGGCGTGATGCTGATGCA-3' |
| P2RX1 | 5'-GGATGGTGCTGGTACGAAACA-3' | 5'-CACTGACACACTGCTGATAAGG-3' |
| GAPDH | 5'-ATCATCAGCAATGCCTCC-3' | 5'-CATCACGCCACAGTTTCC-3' |

**Supplementary Table 2:**

**The information of antibodies**

| Antibody | Company | Dilution ratio |
| --- | --- | --- |
| Bax | Abmart（T40051） | 1:1000 |
| Bcl-2 | Abmart（T40056F） | 1:1000 |
| Cleaved caspase 9 | Selleck（F0326） | 1:1000 |
| Cleaved caspase 3 | Selleck（F0135） | 1:1000 |
| PI3K | Absea（RC6350） | 1:1000 |
| AKT | Abcolnal（A22770） | 1:1000 |
| P-AKT | Cell Signaling Technology（4060T） | 1:1000 |
| P-PI3K | Abcolnal（AP0427） | 1:1000 |
| GAPDH | Abmart（P60037F） | 1:2000 |
| Phospho-CaMKII (Thr286) | Selleck（F0342） | 1:1000 |
| Bad | Selleck（F3233） | 1:1000 |
| Sodium Potassium ATPase | Selleck（F2192） | 1:100000 |
| P2RX1 | Bioss（bs-21358R） | 1:1000 |

**Supplementary Figure 1:**

**CCK8 assay results for KN-62 concentration screening**



Supplementary Figure 1: The total survival rate is depicted as a line graph against the concentration of KN-62. As the concentration and duration of the KN-62 treatment increase, the survival rate experiences a gradual decline. When the concentration exceeds 10 μM, the inhibition rate demonstrates no significant variation. Therefore, 10 μM is selected as the optimal concentration.

**Supplementary Figure 2:**

**CCK8 assay results for imatinib concentration screening**





Supplementary Figure 2: The line graph demonstrates the total survival rate as a function of Imatinib concentration. As the Imatinib concentration and duration increase, the survival rate exhibits a gradual decline. At a concentration of 20 μM, the 24-hour survival rate is approximately 50%. This concentration was selected for subsequent experiments.

**Supplementary Figure 3:**

**P2RX1-Mediated Apoptosis Hypothesis Model**


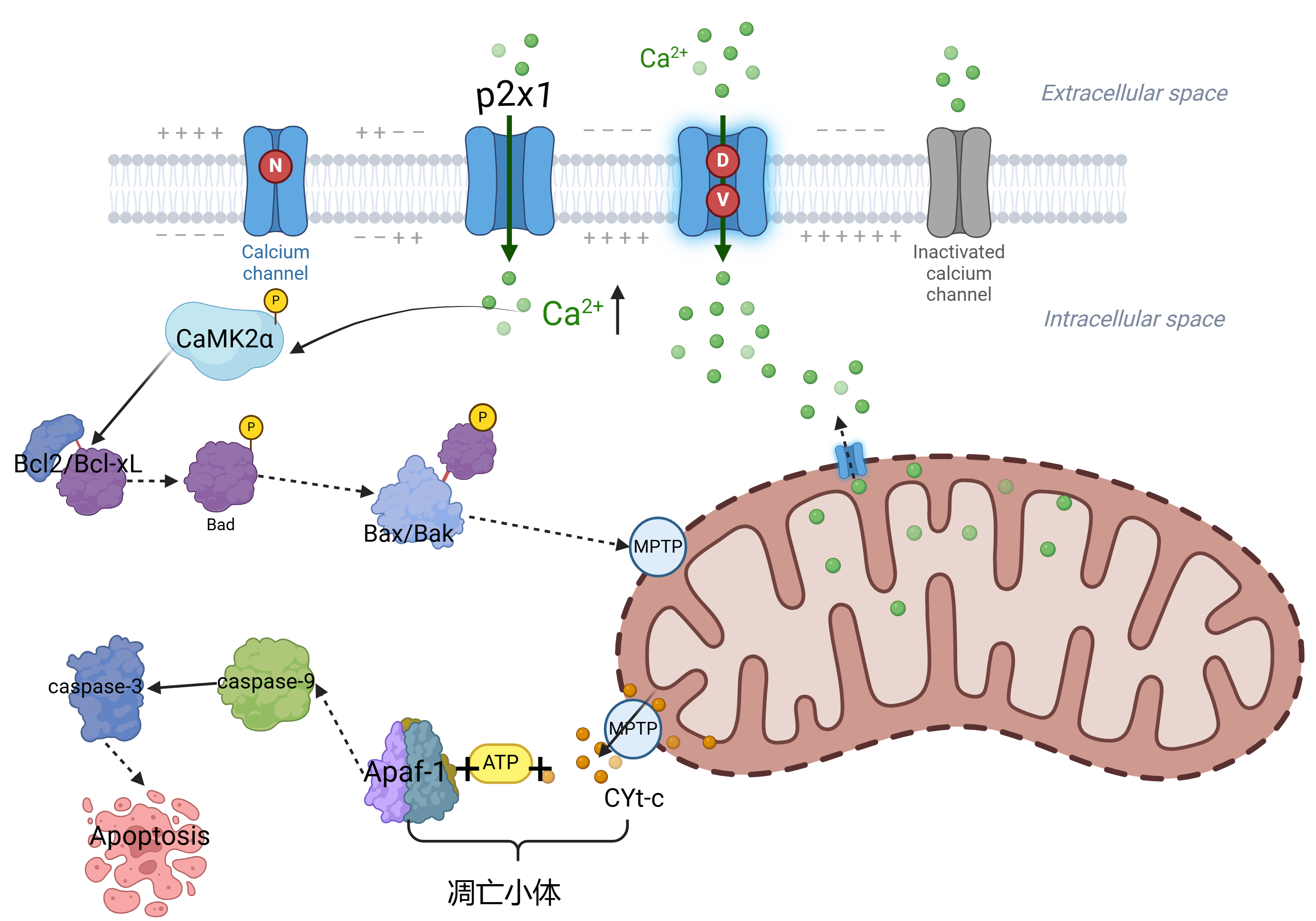


Supplementary Figure 3: Overexpression of P2RX1, acting as a non-selective cation channel on the membrane, increases Ca²⁺ permeability, leading to elevated intracellular calcium concentrations and disruption of calcium homeostasis. This subsequently triggers abnormal activation of CaMKII, which in turn promotes Bad phosphorylation, disrupts the Bcl-2/Bax ratio, opens the mitochondrial permeability transition pore (MPTP), and releases cytochrome c (Cyt c) from mitochondria into the cytoplasm. The released Cyt c participates in the formation of apoptotic bodies, ultimately activating Caspase-9 and Caspase-3 to induce apoptosis.
